# On the relative performance of diagnostic tests

**DOI:** 10.1177/14653125211014901

**Published:** 2021-06-23

**Authors:** Spyridon N Papageorgiou

**Affiliations:** Clinic of Orthodontics and Pediatric Dentistry, Center of Dental Medicine, University of Zurich, Zurich, Switzerland

## A fictional scenario

We are continuing with the clinical research scenario previously discussed in the last two parts of this article series (Papageorgiou, 2020, 2021). Briefly, an orthodontist is trying to retrospectively assess the prevalence of gingival recession among patients within a large university clinic using two different diagnostic tests (Papageorgiou, 2020). These two tests have different diagnostic performances, which are summarised in Table 1.

**Table 1.** Comparative diagnostic performance of the two different diagnostic tests for identifying gingival recessions.

Group	Test 1	Test 2
Sensitivity (%)	80.9	92.7
Specificity (%)	70.5	54.6
Positive predictive value (%)	58.5	51.2
Negative predictive value (%)	87.7	93.5
False negative rate (%)	12.3	6.5
False positive rate (%)	41.5	48.8

However, when applying a diagnostic test to a specific population, it is important to bear in mind that even if its diagnostic performance might remain the same, the prevalence of the disease within the selected sample might vary. Take, as an example, the development of gingival recession on any tooth of the dentition, measured at 15, 18 and 21 years of age. We also want to compare the contribution of orthodontic treatment on the development of gingival recession, so we have two patient groups: (1) one treated orthodontically between 12 and 15 years of age and checked again at 18 and 21 years of age for any recession; and (2) an untreated control group also checked for recession at 15, 18 and 21 years of age. Based on some existing data on this scenario (Renkema et al., 2013), we will assume that the true prevalence of gingival recession for the two patient groups at the corresponding timepoints varies as shown in Table 2.

**Table 2.** Assumed true prevalence of gingival recessions for the various groups and timepoints.

Group	Age: 15 years	Age: 18 years	Age: 21 years
Orthodontically treated (%)	5	15	35
Untreated (%)	0	5	16.7

We will also assume the orthodontist is testing 200 treated patients and 200 untreated controls (equally divided with the two tests; each patient tested only once). In this scenario, which of the following statements is correct, if any?

(a) The observed risk ratio for the development of gingival recession due to orthodontic treatment (as compared with no treatment) will be the same for both diagnostic tests and will be 15%/5% = 3.0 at 18 years of age and 35%/16.7% = 2.1 at 21 years of age.
(b) The observed risk ratio for the development of gingival recession due to orthodontic treatment (as to no treatment) will be different for the two diagnostic tests.
(c) The percentage of true gingival recession that will be missed by the diagnostic test will be different between the two diagnostic tests; this will be independent from the follow-up examination.
(d) The percentage of true gingival recession that will be missed by the diagnostic test will be different between the two diagnostic tests and will vary according to the follow-up examination.
(e) If we now run significantly more tests (500 tests per group/timepoint instead of 100; total of 1000 instead of 200), the percentage of true gingival recession that will be missed by the diagnostic test will be improved (reduced).

## Answers

In order to calculate the risk of recession for each of the investigated groups and timepoints, we first need to calculate how many recessions will be picked up from the test in each instance. Starting from the nominal assumed percentage prevalence, we can multiply this by the number of patients per test and reach the true number of recessions that will be present in the study sample (‘True recessions’ column in Table 3). As can be expected, the results are similar for the two diagnostic tests, since reality is conventionally not affected by which test we might be using.

**Table 3.** Comparative performance of the two theoretical diagnostic tests with 100 tests/patients for each treatment group/timepoint.

Group	Test	Tests	Sensitivity (%)	Recession prevalence (%)			True recessions			Recessions found			Recessions missed (%)		
				T15	T18	T21	T15	T18	T21	T15	T18	T21	T15	T18	T21
Treated	1	100	80.9	5	15	35	5	15	35	4	13	29	20	13	17
Untreated	1	100	80.9	0	5	16.7	0	5	17	0	4	14	-	20	18
Treated	2	100	92.7	5	15	35	5	15	35	5	14	33	0	7	6
Untreated	2	100	92.7	0	5	16.7	0	5	17	0	5	16	-	0	6

T15, 15 years of age; T18, 18 years of age; T21, 21 years of age.

Calculating the recession found by the orthodontist is, however, another story since the test’s diagnostic performance comes into play. We can multiply here the sensitivity of each test with the true recession that exists in a specific patient group per timepoint and arrive at the number of recessions the clinician will be able to identify (‘Recessions found’ column in Table 3). As can be seen, different numbers of recessions are found between the two diagnostic tests in the same group and at the same timepoint (for example 29/100 recessions at treated T21 with test 1 and 33/100 recessions at treated T21 with test 2). Therefore, the observed risk ratio for the development of gingival recession due to orthodontic treatment (as to no treatment) will be different for the two diagnostic tests. At 18 years of age it will be (13/100)/(4/100)=3.25 for diagnostic test 1 and (14/100)/(5/100)=2.80 for diagnostic test 2. Accordingly, at 21 years of age it will be (29/100)/(14/100)=2.07 for diagnostic test 1 and (33/100)/(16/100)=2.06 for diagnostic test 2. So statement (a) is wrong and statement (b) is correct.

As far as the number of true gingival recessions that are missed from our diagnostic test is concerned, it becomes obvious from Table 3 that this differs substantially between the two diagnostic tests. Test 1 has considerably lower sensitivity and therefore misses more recessions than test 2. At the same time, it is obvious that the rate of missed true recessions shows striking fluctuation, especially at T15 and T18, where the prevalence of recession is very low. This is one of the pitfalls of having a diagnostic test that has been developed and validated for use on high-risk symptomatic patients but using it indiscriminately for screening a low-risk asymptomatic general population. So, statement (c) is wrong and statement (d) is correct.

In order to address statement (e), we can look solely at T21 to make things easier (Table 4). Then:

**Table 4.** Comparative performance of the two theoretical diagnostic tests with 100 tests / patients or 500 test / patients for each treatment group / timepoint.

Group	Test	Tests	Sensitivity (%)	Recession prevalence (%)			True recessions			Recessions found			Recessions missed (%)		
				T15	T18	T21	T15	T18	T21	T15	T18	T21	T15	T18	T21
Treated	1	100	80.9	5	15	35	5	15	35	4	13	29	20	13	17
Untreated	1	100	80.9	0	5	16.7	0	5	17	0	4	14	-	20	18
Treated	1	500	80.9	5	15	35	25	75	175	21	61	142	16	19	19
Untreated	1	500	80.9	0	5	16.7	0	25	84	0	21	68	-	16	19
Treated	2	100	92.7	5	15	35	5	15	35	5	14	33	0	7	6
Untreated	2	100	92.7	0	5	16.7	0	5	17	0	5	16	-	0	6
Treated	2	500	92.7	5	15	35	25	75	175	24	70	163	4	7	7
Untreated	2	500	92.7	0	5	16.7	0	25	84	0	24	78	-	4	7

T15, 15 years of age; T18, 18 years of age; T21, 21 years of age.

- The risk ratio for test 1 in the 500-test group is (142/500)/(68/500)=0.284/0.136=2.09, while the risk ratios for the 100-test group is (29/100)/(14/100)=2.07.
- The risk ratio for test 2 in the 500-test group is (163/500)/(78/500)=0.326/0.156=2.09, while the risk ratios for the 100-test group is (33/100)/(16/100)=2.06.

We see, therefore, that relative measures remain mostly stable. A similar finding is seen for the percentage of true recessions missed, where no clear improvement can be seen. Of course, the absolute number of true recessions that are identified is increased in the 500-test group, as is only logical. Additionally, as stated earlier, diagnostic test 2 with the higher sensitivity has significantly lower percentages of true recessions missed by the test. But only increasing the number of tests does not improve the relative performance of the test and statement (e) is wrong.

## References

[bibr1-14653125211014901] Papageorgiou SN (2020) Prudens quaestio in diagnostic studies. Journal of Orthodontics 47: 363–365. 33203336 10.1177/1465312520970946

[bibr2-14653125211014901] Papageorgiou SN (2021) Sampling, testing and interpreting a test’s result. Journal of Orthodontics 48: 88–90. 33843331 10.1177/1465312521995508 PMC8042517

[bibr3-14653125211014901] Renkema AM Fudalej PS Renkema AA Abbas F Bronkhorst E Katsaros C (2013) Gingival labial recessions in orthodontically treated and untreated individuals: a case - control study. Journal of Clinical Periodontology 40: 631–637. 23587032 10.1111/jcpe.12105

